# Multi-locus phylogeny and taxonomy of an unresolved, heterogeneous species complex within the genus *Golovinomyces* (Ascomycota, Erysiphales), including *G. ambrosiae*, *G. circumfusus* and *G. spadiceus*

**DOI:** 10.1186/s12866-020-01731-9

**Published:** 2020-03-05

**Authors:** Peng-Lei Qiu, Shu-Yan Liu, Michael Bradshaw, Suzanne Rooney-Latham, Susumu Takamatsu, Timur S. Bulgakov, Shu-Rong Tang, Jing Feng, Dan-Ni Jin, Temitope Aroge, Yu Li, Li-Lan Wang, Uwe Braun

**Affiliations:** 1 College of Plant Protection, Jilin Agricultural University, Changchun, 130118 Jilin Province People’s Republic of China; 2 School of Environmental and Forest Sciences, University of Washington, Seattle, Washington 98195 USA; 3 California Department of Food & Agriculture, Plany Pest Diagnostic Branch, 3294 Meadowview Road, Sacramento, CA 95832-1448 USA; 4 Faculty of Bioresources, Mie University, Tsu, 514-8507 Japan; 5 Centre for Crop Health, University of Southern Queensland, Toowoomba, QLD 4350 Australia; 6 Russian Research Institute of Floriculture and Subtropical Crops, 2/28 Yana Fabritsiusa Street, Sochi, 354002 Krasnodar Region Russia; 7 Martin Luther University, Institute of Biology, Geobotany and Botanical Garden, Herbarium, Neuwerk 21, 06099 Halle (Saale), Germany

**Keywords:** Erysiphaceae, Powdery mildew, *Heliantheae*, 28S rDNA, ITS, *Golovinomyces latisporus*, *IGS*, *TUB2*, *CHS1*

## Abstract

**Background:**

Previous phylogenetic analyses of species within the genus *Golovinomyces* (Ascomycota*,* Erysiphales), based on ITS and 28S rDNA sequence data, revealed a co-evolutionary relationship between powdery mildew species and hosts of certain tribes of the plant family Asteraceae. *Golovinomyces* growing on host plants belonging to the *Heliantheae* formed a single lineage, comprised of a morphologically differentiated complex of species, which included *G. ambrosiae*, *G. circumfusus*, and *G. spadiceus*. However, the lineage also encompassed sequences retrieved from *Golovinomyces* specimens on other Asteraceae tribes as well as other plant families, suggesting the involvement of a plurivorous species. A multilocus phylogenetic examination of this complex, using ITS, 28S, IGS (intergenic spacer), *TUB2* (beta-tubulin), and *CHS1* (chitin synthase I) sequence data was carried out to clarify the discrepancies between ITS and 28S rDNA sequence data and morphological differences. Furthermore, the circumscription of species and their host ranges were emended.

**Results:**

The phylogenetic and morphological analyses conducted in this study revealed three distinct species named, viz., (1) *G. ambrosiae* emend. (including *G. spadiceus*), a plurivorous species that occurs on a multitude of hosts including, *Ambrosia* spp., multiple species of the *Heliantheae* and plant species of other tribes of Asteraceae including the Asian species of *Eupatorium*; (2) *G. latisporus* comb. nov. (≡ *Oidium latisporum*), the closely related, but morphologically distinct species confined to hosts of the *Heliantheae* genera *Helianthus*, *Zinnia*, and most likely *Rudbeckia*; and (3) *G. circumfusus* confined to *Eupatorium cannabinum* in Europe.

**Conclusions:**

The present results provide strong evidence that the combination of multi-locus phylogeny and morphological analysis is an effective way to identify species in the genus *Golovinomyces*.

## Background

Powdery mildews are obligate biotrophic ascomycetes that occur on a wide range of dicotyledonous and monocotyledonous host plants. The family Erysiphaceae has a nearly worldwide distribution, with the exception of the Antarctic region, and currently comprises around 900 species in 18 genera [1–3]. *Golovinomyces* was originally introduced by Braun [4] as a section of the genus *Erysiphe* (s. lat.) and was later raised to genus rank by Heluta [5]. Braun [6] and Braun and Takamatsu [7] accepted *Golovinomyces* as a distinct genus and established the new tribe Golovinomyceteae*. Golovinomyces* is characterized by having chasmothecia with mycelioid appendages, several, mostly 2-spored asci, an asexual morph with catenescent conidia that lack fibrosin bodies, and mostly nipple-shaped appressoria. *Golovinomyces* currently encompasses 57 species and 5 varieties [1, 8–13]. *Erysiphe cichoracearum* [14] included nearly all of the species that are now assigned to *Golovinomyces*. Blumer [15, 16] split *E. cichoracearum* sensu Salmon [14] into several species but continued to maintain the species *E. cichoracearum* in a very broad sense (covering collections on Asteraceae and on hosts of multiple other plant families). Braun [17] confined *E. cichoracearum* to powdery mildews on hosts of Asteraceae and assigned specimens on hosts belonging to other plant families to *Erysiphe orontii*. Phylogenetic analyses of *Golovinomyces,* based on ITS and 28S rDNA sequence data [18], suggested the co-evolution between *Golovinomyces* species and certain tribes of Asteraceae. Based on these results, Braun and Cook [1] introduced a much narrower species concept for this genus, which included two morphologically differentiated species on hosts belonging to the Heliantheae, viz., *G. ambrosiae* and *G. spadiceus*. However, in more detailed phylogenetic analyses of ITS and 28S rDNA sequences, including *Golovinomyces* species on Asteraceae hosts, Takamatsu et al., [19] found that powdery mildews on hosts of the Heliantheae (previously referred to as *G. ambrosiae* and *G. spadiceus*), on hosts of an Asian species of *Eupatorium* (*G. circumfusus* s. lat.) and on a multitude of other hosts, including those on other plant families, formed a single large, unresolved clade (lineage III in Takamatsu et al., [19]). The taxonomic interpretation of these results posed a serious problem since *G. ambrosiae* and *G. spadiceus*, as circumscribed in Braun and Cook [1], are two morphologically differentiated species. Hence, the resolution within phylogenetic trees based only on ITS sequences was in this case insufficient to discriminate closely allied species. Therefore, most subsequent authors followed the taxonomic treatment in Braun and Cook [1] and recognized *G. ambrosiae* and *G. spadiceus* as separate species within lineage III, based on morphological differences [20–27]. The morphological differences used to differentiate the species include above all, much broader conidia and dimorphic germ tubes belonging to the longitubus pattern within the *Euoidium* type of conidial germination in *G. ambrosiae* than in *G. spadiceus* [1]. Additional research has found *G. spadiceus* to be extremely plurivorous, occurring on hosts of the Heliantheae and other tribes of Asteraceae, e.g., *Aster* and *Chrysanthemum* [19], *Chrysogonum* [28], as well hosts of various other plant families, including *Abelmoschus* (Malvaceae) [29], *Crotalaria* (Fabaceae) [13], *Persicaria* (Polygonaceae) [11, 13, 30], *Solanum* (Solanaceae) [13], and *Verbena* (Verbenaceae) [13]. The taxonomic interpretation of the inclusion of a sequence obtained from a Japanese collection of powdery mildew on *Eupatorium chinense* in lineage III [19] caused an additional problem and raised the question whether the name *G. circumfusus*, originally described from Europe on *Eupatorium cannabinum*, is included in this species complex.

The purpose of the present study was to clarify and resolve the taxonomy of this *Golovinomyces* complex using a multilocus approach, based on ITS, 28S, IGS, *TUB2* and *CHS1* DNA sequences. Multi-gene analyses are currently the method of choice to analyze phylogenetically and taxonomically difficult complexes of plant pathogenic fungi, including *Colletotrichum* spp. [31, 32]. However, there is minimal multilocus data for the powdery mildews currently available. Most of the research involves the intraspecific genetic diversity in species such as *Blumeria graminis* [33, 34], *Erysiphe japonica* [35], *E. necator* [36, 37], *Podosphaera xanthii* [38] and *Golovinomyces orontii* [39]. Recently, the geographic and temporal distributions of four genotypes found in *E. gracilis* var. *gracilis* were studied based on a combination of data from the ITS, 28S rDNA and IGS regions [40]. Comprehensive applications of multilocus approaches to solve complex taxonomic-phylogenetic problems connected with the species level classification of the powdery mildews are still lacking. The present study is the first to use a multilocus approach to solve species distinction issues within the Erysiphales. An additional issue regarding the taxonomic conclusions drawn from phylogenetic results is also addressed in this study. Older taxonomic names are often available, but the application and allocation of such names are usually problematic. Because species names are based on their type collections, epitypifications, with appropriate new material, and ex-epitype sequences tend to be the main method to overcome these obstacles and to determine the application of older names. During the current study, this issue was addressed using international collaboration.

## Methods

### Sampling

A total of 69 specimens belonging to *Golovinomyces ambrosiae*, *G. circumfusus*, and *G. spadiceus* were examined, including 39 samples collected in China in recent years and 30 additional specimens from Germany, Japan, Russia, Switzerland, and the USA. Furthermore, eight specimens, consisting of three samples of *G. magnicellulatus*, three samples of *Neoërysiphe galeopsidis*, a sample of *Arthrocladiella mougeotii* and a sample of *Erysiphe kenjiana*, were used for phylogenetic analyses in this study. All of the plant materials used in this study were collected in the public gardens with Latin names or some are common ornamental plants which were identified by ourselves. Among the 69 specimens, ISC-F-0076752, ISC-F-0076753, and ISC-F-0076754 were deposited in the Herbarium of Iowa State University Fungi of Iowa, and the rest voucher specimens were deposited in the Herbarium of Mycology of Jilin Agricultural University. Names of the host plants, fungal species, locations and years of collection, voucher numbers and newly sequenced multi-gene accession numbers for the nucleotide sequence database (GenBank) in this study are given in Table 1.

**Table 1:** Information of powdery mildew vouchers studied in this paper

Species	Host	Location	Year of collection	Voucher ^a^	GenBank accessions No. ^b^				
					ITS	28S	IGS	*TUB2*	*CHS1*
*Arthrocladiella mougeotii*	*Lycium chinense*	Beijing, China	2018	HMJAU-PM91837	MK452607	MK452680	–	–	–
*Erysiphe kengiana*	*Ulmus pumila*	Changchun, Jilin province, China	2017	HMJAU-PM91841	MK452611	MK452684	–	MK452458	–
*Golovinomyces ambrosiae*	*Aster novi-belgii*	Changchun, Jilin province, China	2017	HMJAU-PM91804	MK452575	MK452648	MK452501	MK452460	MK452410
*G. ambrosiae*	*A. novi-belgii*	Changchun, Jilin province, China	2018	HMJAU-PM91805	MK452576	MK452649	MK452502	MK452461	MK452411
*G. ambrosiae*	*A. novi-belgii*	Dunhua, Jilin province, China	2018	HMJAU-PM91806	MK452577	MK452650	MK452503	MK452462	MK452412
*G. ambrosiae*	*A. novi-belgii*	Dunhua, Jilin province, China	2018	HMJAU-PM91807	MK452578	MK452651	MK452504	MK452463	MK452413
*G. ambrosiae*	*A. novi-belgii*	Changchun, Jilin province, China	2017	HMJAU-PM91808	MK452579	MK452652	MK452505	MK452464	MK452414
*G. ambrosiae*	*Ageratina ligustrina*	Sochi city, Krasnodar region, Russia	2018	ERY015	MK452643	MK452717	MK452570	–	–
*G. ambrosiae*	*Ambrosia artemisiifolia*	Mudanjiang, Heilongjiang, China	2017	HMJAU-PM91809	MK452580	MK452653	MK452506	MK452465	MK452415
*G. ambrosiae*	*A. artemisiifolia*	Changchun, Jilin province, China	2018	HMJAU-PM91810	MK452581	MK452654	MK452507	MK452466	MK452416
*G. ambrosiae*	*A. artemisiifolia*	Tonghua, Jilin province, China	2018	HMJAU-PM91811	MK452582	MK452655	MK452508	MK452467	MK452417
*G. ambrosiae*	*A. artemisiifolia*	Tonghua, Jilin province, China	2018	HMJAU-PM91812	MK452583	MK452656	MK452509	–	MK452418
*G. ambrosiae*	*A. artemisiifolia*	Guthrie County, Iowa, USA	1987	ISC-F-0076752	–	–	MK452567	–	–
*G. ambrosiae*	*A. trifida*	Guthrie County, Iowa, USA	1987	ISC-F-0076754	–	MK452715	–	–	–
*G. ambrosiae*	*A. trifida*	Guthrie County, Iowa, USA	1997	ISC-F-0076753	–	–	MK452568	–	–
*G. ambrosiae*	*A. trifida*	Siping, Jilin province, China	2018	HMJAU-PM91813	MK452584	MK452657	MK452510	MK452468	MK452419
*G. ambrosiae*	*A. trifida*	Changchun, Jilin province, China	2018	HMJAU-PM91814	MK452585	MK452658	MK452511	MK452469	MK452420
*G. ambrosiae*	*A. trifida*	Anshan, Liaoning, China	2018	HMJAU-PM91815	MK452586	MK452659	MK452512	MK452470	MK452421
*G. ambrosiae*	*A. trifida*	Shenyang, Liaoning, China	2018	HMJAU-PM91816	MK452587	MK452660	MK452513	–	MK452422
*G. ambrosiae*	*Dahlia pinnata*	Dandong, Liaoning, China	2012	HMJAU-PM91817	MK452588	MK452661	MK452514	–	–
*G. ambrosiae*	*D. pinnata*	Changchun, Jilin province, China	2017	HMJAU-PM91818	MK452589	MK452662	MK452515	MK452471	MK452423
*G. ambrosiae*	*D. pinnata*	Changchun, Jilin province, China	2017	HMJAU-PM91819	MK452590	MK452663	MK452516	MK452472	MK452424
*G. ambrosiae*	*D. pinnata*	Changchun, Jilin province, China	2018	HMJAU-PM91820	MK452591	MK452664	MK452517	MK452473	MK452425
*G. ambrosiae*	*D. pinnata*	Siping, Jilin province, China	2018	HMJAU-PM91821	MK452592	MK452665	MK452518	MK452474	MK452426
*G. ambrosiae*	*D. pinnata*	Panzhihua, Sichuan, China	2018	HMJAU-PM91822	MK452593	MK452666	MK452519	MK452475	MK452427
*G. ambrosiae*	*Dahlia* sp.	Yolo Co. CA, USA	2018	MVAP50000445	MK452632	MK452705	MK452557	–	–
*G. ambrosiae*	*Dahlia* sp.	Santa Barbara Co. CA, USA	2018	LM0P03825217–1	MK452637	MK452710	MK452562	–	MK452457
*G. ambrosiae*	*Dahlia* sp.	Seattle Washington, USA	2018	HMJAU-PM91854	MK452641	MK452714	MK452566	–	–
*G. ambrosiae*	*Eupatorium japonicum*	Aichi, Nagoya-shi, Japan	2001	MUMH4142	MK452621	MK452694	MK452546	–	–
*G. ambrosiae*	*E. makinoi*	Katashina-mura, Gunma, Japan	2002	MUMH4143	MK452622	MK452695	MK452547	–	–
*G. ambrosiae*	*E. makinoi*	Tochigi, Sano, Japan	2002	MUMH4424	MK452623	MK452696	MK452548	–	–
*G. ambrosiae*	*E. makinoi*	Okayama-shi, Okayama, Japan	2003	MUMH4794	MK452625	MK452698	MK452550	–	–
*G. ambrosiae*	*E. makinoi*	Shiga, Maibara, Japan	2017	MUMH7129	MK452624	MK452697	MK452549	–	–
*G. ambrosiae*	*E. japonicum*	Mie, Tsu-shi, Japan	2018	HMJAU-PM91855	MK452626	MK452699	MK452551	MK452496	MK452453
*G. ambrosiae*	*Leucanthemum maximum*	Changchun, Jilin province, China	2016	HMJAU-PM91836	KX987303	MF612182	MK452533	MK389490	MK389489
*G. ambrosiae*	*Zinnia elegans*	Chengdu, Sichuan, China	2016	HMJAU-PM91842	MK452612	MK452685	MK452537	MK452487	MK452444
*G. ambrosiae*	*Z. elegans*	Changchun, Jilin province, China	2017	HMJAU-PM91843	MK452613	MK452686	MK452538	MK452488	MK452445
*G. ambrosiae*	*Z. elegans*	Changchun, Jilin province, China	2017	HMJAU-PM91844	MK452614	MK452687	MK452539	MK452489	MK452446
*G. ambrosiae*	*Z. elegans*	Changchun, Jilin province, China	2017	HMJAU-PM91845	MK452615	MK452688	MK452540	MK452490	MK452447
*G. ambrosiae*	*Z. elegans*	Changchun, Jilin province, China	2018	HMJAU-PM91846	MK452616	MK452689	MK452541	MK452491	MK452448
*G. ambrosiae*	*Z. elegans*	Siping, Jilin province, China	2018	HMJAU-PM91847	MK452617	MK452690	MK452542	MK452492	MK452449
*G. ambrosiae*	*Z. elegans*	Tonghua, Jilin province, China	2018	HMJAU-PM91848	MK452618	MK452691	MK452543	MK452493	MK452450
*G. ambrosiae*	*Z. elegans*	Siping, Jilin province, China	2018	HMJAU-PM91849	MK452619	MK452692	MK452544	MK452494	MK452451
*G. ambrosiae*	*Z. elegans*	Santa Barbara Co. CA, USA	2018	LM0P06825217–3	MK452633	MK452706	MK452558	–	MK452456
*G. ambrosiae*	*Z. elegans*	Yolo Co. CA, USA	2018	MVAP50000452	MK452634	MK452707	MK452559	–	–
*G. circumfusus*	*Eupatorium cannabinum*	Altmark, Sachsen-Anhalt, Germany	2000	GLM49501	MK452630	MK452703	MK452553	–	–
*G. circumfusus*	*E. cannabinum*	Landkreis Ostprignitz-Ruppin, Brandenburg, Germany	2006	GLM74796	MK452629	MK452702	MK452554	–	–
*G. circumfusus*	*E. cannabinum*	Spreewald, Brandenburg, Germany	2016	HAL 3300 F	MK452628	MK452701	MK452555	MK452459	MK452455
*G. latisporus*	*Helianthus annuus*	Changchun, Jilin province, China	2017	HMJAU-PM91830	MK452601	MK452674	MK452527	MK452483	MK452435
*G. latisporus*	*H. annuus*	Changchun, Jilin province, China	2017	HMJAU-PM91828	MK452599	MK452672	MK452525	MK452481	MK452433
*G. latisporus*	*H. annuus*	Yichun, Heilongjiang, China	2017	HMJAU-PM91829	MK452600	MK452673	MK452526	MK452482	MK452434
*G. latisporus*	*H. annuus*	Tonghua, Jilin province, China	2018	HMJAU-PM91831	MK452602	MK452675	MK452528	MK452484	MK452436
*G. latisporus*	*H. annuus*	Panzhihua, Sichuan, China	2018	HMJAU-PM91832	MK452603	MK452676	MK452529	MK452485	MK452437
*G. latisporus*	*H. tuberosus*	Chongqing, China	2014	HMJAU-PM91823	MK452594	MK452667	MK452520	MK452476	MK452428
*G. latisporus*	*H. tuberosus*	Shangqiu, Henan, China	2016	HMJAU-PM91824	MK452595	MK452668	MK452521	MK452477	MK452429
*G. latisporus*	*H. tuberosus*	Changchun, Jilin province, China	2017	HMJAU-PM91825	MK452596	MK452669	MK452522	MK452478	MK452430
*G. latisporus*	*H. tuberosus*	Changchun, Jilin province, China	2017	HMJAU-PM91826	MK452597	MK452670	MK452523	MK452479	MK452431
*G. latisporus*	*H. tuberosus*	Changchun, Jilin province, China	2017	HMJAU-PM91827	MK452598	MK452671	MK452524	MK452480	MK452432
*G. latisporus*	*H. tuberosus*	Shakhty city, Rostov region, Russia	2018	ERY057	MK452642	MK452716	MK452569	–	–
*G. latisporus*	*H. tuberosus*	Shakhty city, Rostov region, Russia	2018	ERY061	MK452644	MK452718	MK452571	–	–
*G. latisporus*	*H. tuberosus*	Shakhty city, Rostov region, Russia	2018	ERY081	MK452645	MK452719	MK452572	–	–
*G. latisporus*	*H. tuberosus*	Shakhty city, Rostov region, Russia	2018	ERY094	MK452646	MK452720	MK452573	–	–
*G. latisporus*	*H. tuberosus*	Novoshakhtinsk city, Rostov region, Russia	2018	ERY152	MK452647	MK452721	MK452574	–	–
*G. latisporus*	*H. annuus*	Nyon, Vaud, Switzerland	2018	HAL 3299 F	MK452627	MK452700	MK452552	MK452497	MK452454
*G. latisporus*	*H. annuus*	Solano Co. CA, USA	2018	MVAP50000419	MK452635	MK452708	MK452560	MK452498	–
*G. latisporus*	*H. annuus*	Santa Barbara Co. CA, USA	2018	LM0P03825217–2	MK452636	MK452709	MK452561	MK452499	–
*G. latisporus*	*H. annuus*	Seattle Washington, USA	2018	HMJAU-PM91853	MK452640	MK452713	MK452565	–	–
*G. latisporus*	*H. mollis*	Seattle Washington, USA	2018	HMJAU-PM91851	MK452638	MK452711	MK452563	–	–
*G. latisporus*	*Helianthus* sp.	Seattle Washington, USA	2018	HMJAU-PM91852	MK452639	MK452712	MK452564	MK452500	–
*G. latisporus*	*Zinnia angustifolia*	Potsdam, Brandenburg, Germany	2008	HAL 2338 F	MK452631	MK452704	MK452556	–	–
*G. latisporus*	*Z. elegans*	Panzhihua, Sichuan, China	2018	HMJAU-PM91850	MK452620	MK452693	MK452545	MK452495	MK452452
*G. magnicellulatus*	*Physalis alkekengi*	Yichun, Heilongjiang, China	2017	HMJAU-PM91838	MK452608	MK452681	MK452535	–	MK452441
*G. magnicellulatus*	*P. alkekengi*	Changchun, Jilin province, China	2017	HMJAU-PM91839	MK452609	MK452682	MK452536	–	MK452442
*G. magnicellulatus*	*P. alkekengi*	Changchun, Jilin province, China	2018	HMJAU-PM91840	MK452610	MK452683	MK452534	MK452486	MK452443
*Neoërysiphe galeopsidis*	*Leonurus artemisia*	Beijing, China	2018	HMJAU-PM91833	MK452604	MK452677	MK452530	–	MK452438
*N. galeopsidis*	*L. artemisia*	Beijing, China	2018	HMJAU-PM91834	MK452605	MK452678	MK452531	–	MK452439
*N. galeopsidis*	*L. artemisia*	Changchun, Jilin province, China	2017	HMJAU-PM91835	MK452606	MK452679	MK452532	–	MK452440

^a^*HMJAU* Herbarium of Mycology of Jilin Agricultural University; *HAL* Herbarium of Halle University; *GLM* Herbarium of Senckenberg Museum für Naturkunde Görlitz; *MUMH* Mie University Mycological Herbarium; *ERY* herb. Bulgakov; *LM and MVAP* herb. S. Rooney Latham; *ISC* Iowa State University. The specimens GLM74796, GLM49501 (herbarium GLM, Görlitz, Germany), HAL 2338 F, HAL 3299 F, and HAL 3300 F (herbarium HAL, Halle [Saale], Germany) were supplied by Uwe Braun. The specimens MUMH4142, MUMH4143, MUMH4424, MUMH7129, MUMH4794, and HMJAU-PM91855 (herbarium MUMH, Mie, Japan) were provided by Susumu Takamatsu. The specimens MVAP50000419, MVAP50000445, MVAP50000452, LM0P03825217–1, LM0P03825217–2, and LM0P06825217–3 were supplied by Suzanne Latham-Rooney. The specimens HMJAU-PM91851, HMJAU-PM91852, HMJAU-PM91853, HMJAU-PM91854, ISC-F-0076752, ISC-F-0076753, and ISC-F-0076754 were supplied by Michael Bradshaw; and ERY015, ERY057, ERY061, ERY057, ERY081, ERY094 and ERY152 by Timur S. Bulgakov

^b^“–” means failed to get sequence

### Morphological examinations

For microscopic examinations, fresh samples were mounted in sterile water, and dried specimens, scraped from the leaf surface with a clean scalpel, were mounted in a drop of lactic acid on a microscope slide. Slides were examined using light microscopy with the total magnification at 200 and 400 (Zeiss Axio Scope A1, Germany). Fresh conidia were examined for the presence or absence of fibrosin bodies. A minimum of 30 measurements were made of asexual and sexual fungal structures. Germination of conidia was examined following the method of Hirata [41].

### Molecular techniques and phylogenetic analyses

Whole-cell DNA was extracted from chasmothecia or conidia and mycelia by the Chelex-100 method [42, 43]. In the USA, whole-cell DNA was extracted from chasmothecia or conidia (for the herbarium specimens: ISC-F-0076752, ISC-F-0076753, and ISC-F-0076754) with the DNeasy plant mini kit (Qiagen, Hilden, Germany), following the manufacturer’s protocol. Five genomic regions (ITS, 28S rDNA, IGS, *TUB2*, *CHS1*) were selected for phylogenetic analyses. The sequences and references of primers used to amplify these regions are shown in Table 2. For the *TUB2* gene, primers TubF1/TubR1 were designed based on scaffold_4647 in genome of *Erysiphe necator* (GenBank ID: JNVN00000000.1) [47], contig c9894 in genome of *E.pisi* (GenBank ID: CACM00000000) and *TUB2* sequence of *G. orontii* (KR815663) from Pirondi et al., [39]. For the *CHS1* region, primers gCS1a1/gCS1b were designed based on the *CHS1* sequences of AF188934 from *Blumeria graminis* [48], KJ698665 from *Podosphaera xanthii* [38], scaffold_1559 in the genome of *E. necator* (GenBank ID: JNVN00000000.1), contig1307 in the genome of *G. orontii* from the Joint Genome Institute (JGI) (Project ID: 1055997), and contig c7151 in the genome of *E. pisi* (GenBank ID: CACM00000000).

**Table 2:** Primer sets for multilocus sequence typing (MLST) analysis of *Golovinomyces* in this study

DNA regions	Primer	Primer sequences (5′ → 3′)	Annealing temperature (°C)	Amplicon size (bp)	Reference
ITS	ITS5 ITS4	GGAAGTAAAAGTCGTAACAAGG TCCTCCGCTTATTGATATGC	52	600	[44]
28S rDNA	LSU1 LSU2	ACCCGCTGAACTTAAGCATA CCTTGGTCCGTGTTTCAAGA	52	500	[45]
IGS	IGS-12a NS1R	AGTCTGTGGATTAGTGGCCG GAGACAAGCATATGACTAC	52	400	[46]
*TUB2*	TubF1 TubR1	AGGTTCACCTCCAGACTGG CCAGCACGAACAGCATCCAT	52	450	This study
*CHS1*	gCS1a1 gCS1b	GGTGCATTCTCGGCATATCG CGTCACCCTTGGTGCCCCAAG	52	1000	This study

To obtain sufficient DNA for sequencing, the DNA regions of *TUB2* and *CHS1* were amplified by two rounds of PCR with the same primer set. All PCR reactions were conducted in 25 μL volumes. The reaction components were 2.5 μL 10 × PCR Buffer (Mg^2+^ plus) (TaKaRa, Japan), 2 μL dNTP Mixture (10 mM total, 2.5 mM each), 1 μL each primer (20 ng/μL), 2 μL of total genomic DNA, 0.1 μL Taq polymerase (TaKaRa, Japan) (5 U/μL) and sterile ddH_2_O up to a final volume of 25 μL. The PCR reactions were conducted under the following thermal cycling conditions: an initial denaturation step of 5 min at 95 °C, 35 cycles of 1 min at 94 °C, followed by 30 s at 52 °C for annealing, and 2 min at 72 °C for extension, and a final extension for 8 min at 72 °C. A negative control that lacked template DNA was included in each set of reactions. PCR products were subjected to electrophoresis in a 1.2% agarose gel in 0.5× TBE buffer. The amplified DNA products were purified using Mag-MK PCR Products Purification Kit following the protocol of the manufacturer. Amplicons were sequenced in both directions with the same PCR primers using direct sequencing in a 3730xl DNA Analyzer (Applied Biosystems) by Sangon Biotech (Shanghai, China). The sequence reactions were conducted using the BigDye™ Terminator v3.1 Cycle Sequencing Kit (Applied Biosystems) following instructions of the manufacturer.

The reaction components for the PCR conducted at the University of Washington were 5 uL AllTaq PCR Buffer (Qiagen, Germany), 0.5 uL dNTP mixture, 0.25 μL of each primer (100 uM), 2 μL of total genomic DNA, 0.5 μL, Taq Polymerase (Qiagen, Germany) and sterile ddH_2_O up to a final volume of 25 μL. DNA was purified by isopropanol precipitation. These sequences [(The 28S rDNA sequence from ISC-F-0076754 and IGS sequences from ISC-F-0076752 and ISC-F-0076753] were manually trimmed using Geneious version 11.0.2 (https://www.geneious.com) and deposited in GenBank.

All other new sequences obtained in the present study were edited by DNAMAN version 6.0 and BioEdit Sequence Alignment Editor version 7.0, and then deposited in GenBank (Table 1). The ITS, 28S, IGS, *TUB2* and *CHS1* sequences were respectively aligned by ClastalW. Furthermore, a multilocus sequences alignment datasets file (ITS+28S + IGS + *TUB2* + *CHS1*) including 40 strains from Table 1 was also used for phylogenetic analyses. The six alignments were further refined manually in MEGA 7.0 [49] and deposited in TreeBASE (http://www.treebase.org/) under the Accession No. of S24404 (http://purl.org/phylo/treebase/phylows/study/TB2:S24404). Phylogenetic trees were obtained from the sequence data using maximum parsimony (MP) in PAUP 4.0b [50]. The MP analyses were performed with heuristic search option using the tree bisection reconnection (TBR) algorithm with 100 random sequence additions to find the global optimum tree. All sites were treated as unordered and unweighted, with gaps treated as missing data. The strength of the internal branches of the resulting trees were tested with bootstrap (BS) analysis using 1000 replications. Tree scores, including tree length, consistency index (CI), retention index (RI), and rescaled consistency index (RC), were also calculated. Five phylogenetic trees were generated based on the ITS, 28S, IGS, *TUB2* and *CHS1* nucleotide sequences.

## Results

### Phylogenetic analyses

Parsimoniuous trees were separately constructed based on sequences of five gene regions and their combination and the numerical data including the number of taxa and characters are shown in Table 3. The information of outgroup taxon for each phylogenetic tree was also included in Table 1. The phylogenetic trees based on the ITS and 28S rDNA sequences were topologically congruent and indicated that *G. ambrosiae* complex on many Asteraceae plants, including *Eupatorium* spp. from Japan, formed a single clade with 100 and 99% bootstrap support, respectively (see Additional files 1, 2: Figure S1, S2).

**Table 3:** Information of the data matrices and the respective trees based on five individual gene regions

DNA region	ITS	28S	IGS	*TUB2*	*CHS1*	ITS+28S + IGS + *TUB2* + *CHS1*
Number of sequences	74	75	74	44	49	40
Number of characters	509	639	393	432	968	2931
Number of parsimony-uninformative characters	50	26	1	112	22	182
Number of parsimony-informative characters	108	41	104	30	107	102
Tree length	228	87	133	164	154	305
Consistency index (CI)	0.8684	0.8621	0.8947	0.9512	0.9156	0.9902
Retention index (RI)	0.9242	0.9250	0.9595	0.9175	0.9698	0.9855
Rescaled consistency index (RC)	0.8026	0.7974	0.8585	0.8728	0.8879	0.9758

**Fig. 1:**
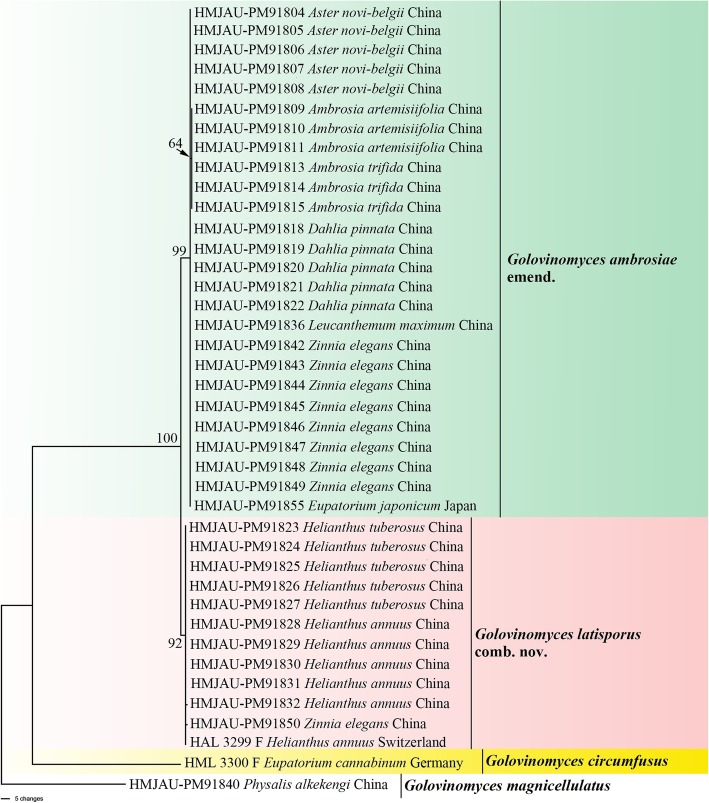
Phylogenetic analysis based on the combined sequence datasets of ITS+28S rDNA+IGS + *TUB2* + *CHS1* of the *Golovinomyces ambrosiae* complex and *G. circumfusus*. The tree was constructed based on 40 strains from genus *Golovinomyces*. *G. magnicellulatus* (voucher: HMJAU-91840) was used as outgroup. Bootstrap values based on 1000 replications are indicated above/below the branches

*Golovinomyces circumfusus* on *E. cannabinum* from Germany did not form a monophyletic group with *G. ambrosiae* complex in all phylogenies (see Additional file 1–5: Figure S1–S5 and Fig. 1). The phylogenetic tree of IGS was similar to ITS tree, with the *G. ambrosiae* complex formed a single clade with 100% bootstrap support based on the individual genes (see Additional file 3: Figure S3). However, the isolates from *Helianthus* spp. and some *Zinnia* spp. differed by one base from isolates on other host genera, and forming a subclade with 64% bootstrap support (see Additional file 3: Figure S3 pink clade). The *G. ambrosiae* complex included two groups, one identified as *G. ambrosiae* emend. (see Additional file 3: Figure S3 green clade) and the other as *G. latisporus* comb. nov. (see Additional file 3: Figure S3 pink clade), based on the phylogenetic analysis of the IGS. The *G. ambrosiae* complex in *TUB2* and *CHS1* trees was divided into two subgroups, viz. *G. ambrosiae* emend., including *G. spadiceus* with 91 and 85% bootstrap support respectively (see Additional files 4, 5: Figure S4, S5 green clade), and *G. latisporus* comb. nov. with 70 and 78% bootstrap support respectively (see Additional files 4, 5: Figure S4, S5 pink clade). In the *G. ambrosiae* emend. Clade the sequences of *CHS1* from isolates on *Ambrosiae artemisiifolia* and *A. trifida* differed by one base from isolates on other hosts. *Golovinomyces ambrosiae* emend. is a plurivorous species that occurs on a multitude of hosts including, *Ambrosia* spp., multiple species from the Heliantheae and plant species of other tribes of Asteraceae including the Asian species of *Eupatorium*. *Golovinomyces latisporus* comb. nov. was confined to hosts of the Heliantheae genera *Helianthus* and *Zinnia*.

Furthermore, the bootstrap values of clades *G. ambrosiae* emend. and *G. latisporus* comb. nov. (BS = 99 and 92% respectively) in combined analysis (see Fig. 1) were higher than in other trees that were constructed based on separate genes. *Golovinomyces circumfusus* on *E. cannabinum* from Europe, forming a single clade, represented a separate species based on the combined data analysis (see Fig. 1).

### Taxonomy

**Fig. 2:**
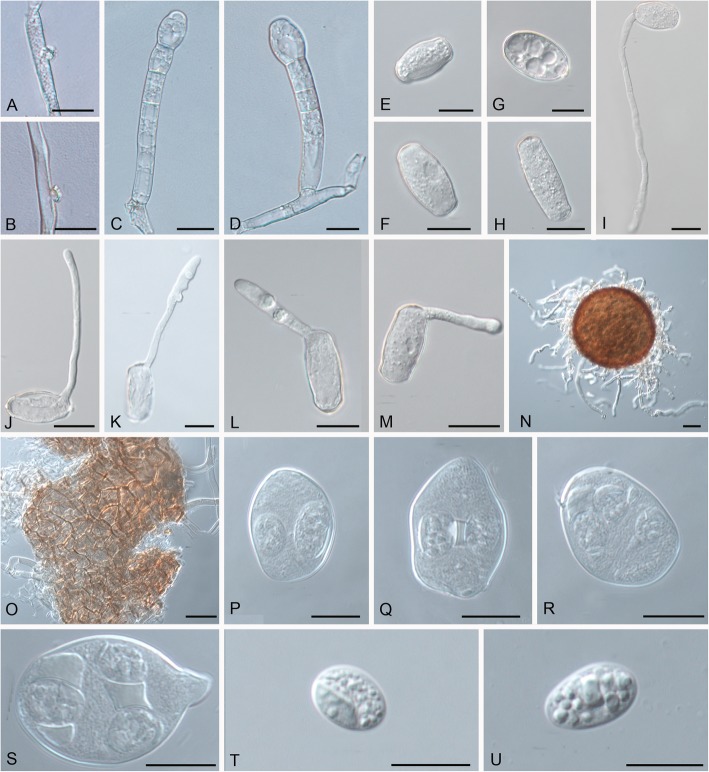
*Golovinomyces ambrosiae* (HMJAU-PM91814 ex *Ambrosia trifida*). **a.** Nipple-shaped hyphal appressorium. **b.** Slightly crenulate hyphal appressorium. **c–d.** Conidiophores. **e–h.** Conidia. **i**–**m.** Conidial germination. **n.** Chasmothecium. **o.** Peridium cells of Chasmothecium. **p–q.** Asci with two ascospores. **r–s.** Asci with three ascospores. **t–u.** Ascospores. Scale bars = 20 μm

***Golovinomyces ambrosiae*** (Schwein.) U. Braun & R.T.A. Cook, in Cook & Braun, Mycol. Res. 113: 628 (2009). Figure 2.

≡ *Erysiphe ambrosiae* Schwein., Trans. Amer. Philos. Soc., N.S., 4: 270 (1834).

= *Erysiphe spadicea* Berk. & M.A. Curtis, Grevillea 4: 159 (1876).

≡ *Golovinomyces spadiceus* (Berk. & M.A. Curtis) U. Braun, in Braun & Cook, CBS Biodiversity Series 11: 329 (2012).

= *Erysphe cichoracearum* f. *ambrosiae* Jacz., Karm. Opred. Grib., Vip. 2. Muchn.-rosj. Griby (Leningrad): 186 (1927).

= *Erysiphe cichoracearum* f. *xanthii* Jacz., Karm. Opred. Grib., Vip. 2. Muchn.-rosj. Griby (Leningrad): 212 (1927).

= *Oidium acanthospermi* Chidd., Lloydia 18: 46 (1955).

≡ *Acrosporium acanthospermi* (Chidd.) Subram., Hyphomycetes (New Delhi): 835 (1971).

= *Oidium lagasceae* Chidd., Lloydia 18: 47 (1955).

≡ *Acrosporium lagasceae* (Chidd.) Subram., Hyphomycetes (New Delhi): 836 (1971).

= *Oidium parthenii* Satyapr. & Ushar., Curr. Sci. 50: 1081 (1981).

= *E. cichoracearum* var. *transvaalensis* G.J.M. Gorter & Eicker, S. Afr. J. Bot. 2(2): 130 (1983).

≡ *Golovinomyces cichoracearum* var. *transvaalensis* (G.J.M. Gorter & Eicker) U. Braun, Schlechtendalia 3: 51 (1999).

= *Oidium blainvilleae* Bappamm., Hosag. & Udaiyan, New Botanist 22: 117 (1995).

= *Erysiphe cichoracearum* auct. p.p.

= *Golovinomyces cichoracearum* auct. p.p.

*Literature*: Braun and Cook ([1], p. 330), Dugan [51], Takamatsu et al., [19], Khodaparast [52], Arzanlou et al., [53], Meeboon et al., ([11], p. 212), Moparthi et al., [29, 54], Trigano et al., [28], Braun et al., [13].

*Illustrations*: Bappammal et al., ([55], p. 110, Fig. 26, 115, Fig. 35), Nomura ([56], p. 182, Fig. 241), Braun and Cook ([1], p. 330, Fig. 359), Meeboon et al., ([11], p. 211, Figs. 9–11).

*Exsiccatum*: Seym. & Earle, Econ. Fungi 321.

*Description*: *Mycelium* amphigenous and caulicolous, white, in small to moderately large patches, confluent, sometimes covering entire leaves, persistent or almost so; *hyphae* 2–9 μm wide, thin-walled, smooth, hyaline, in old infections hyphae around ascomata sometimes turning brown; *hyphal appressoria* solitary, sometimes several per hyphal cell, nipple-shaped, occasionally slightly crenulate or irregular, 3–8 μm diam.; *conidiophores* erect, arising from the upper surface of the hyphal mother cell and usually towards one end of it; *foot-cells* cylindrical, straight, rarely slightly flexuous, 30–80 × 9–15 μm, followed by 1–3 shorter cells, forming catenescent conidia; *conidia* ellipsoid-ovoid, doliiform-subcylindrical, 25–40 × 14–20(− 24) μm, length/width ratio 1.5–2; *conidial germination* of the *Euoidium* type. *Chasmothecia* amphigenous, occasionally caulicolous, scattered to gregarious, 80–140 μm diam., rarely larger; *peridium cells* irregularly shaped, polygonal to daedaleoid, 8–30 μm diam., walls of the cells up to 2 μm wide; *appendages* numerous, mostly arising from the lower half, mycelioid, usually unbranched, 0.2–1.5 times as long as the chasmothecial diam., mostly shorter than the diam, (3–)4–8(− 10) μm wide, at first hyaline, later yellowish to medium brown throughout or paler towards the tips, septate, walls thin, smooth or almost so; *asci* numerous, mostly (5–)8–15, obovoid-saccate, 40–70 × 25–35(− 40) μm, almost sessile or short-stalked, wall thin, up to 1 μm thick, 2(− 3)-spored; *ascospores* broad ellipsoid-ovoid, 15–25(− 28) × 10–15(− 18) μm, colorless.

*Material examined*: additional collections used for molecular analyses (see Table 1); USA, Pennsylvania, Lehigh & Northampton, Bethleham, on leaves of *Ambrosia* sp. (Asteraceae), 1826, L. von Schweinitz, PH 62362, **holotype** of *E. ambrosiae*; USA, South Carolina, on leaves of *Xanthium* sp. (Asteraceae), ex herb. M.J. Berkeley, No. 2984, K(M) 164,976, **holotype** of *E. spadiceus*. JAPAN, Mie Pref., Tsu, on leaves of *Xanthium strumarium* (Asteraceae), 12 Nov. 1997, S. Takamatsu, TSU-MUMH 413 (reference material for *Erysiphe spadicea* with ex-reference material sequence – AB077644, see Braun et al. 2019). USA, Iowa, Guthrie County, Sheeder Prairie State Preserve, on leaves of *Ambrosia trifida*, 12 Aug. 1997, Lois H. Tiffany, ISC-F-0076753, **epitype** of *Erysiphe ambrosiae* (designated here, MycoBank MBT385758).

*Host range and distribution* (see [1, 13]): widespread in Asia, Australia, Europe and North America, on species of numerous host genera belonging to the families Asteraceae (*Acanthospermum*, *Ambrosia*, *Aster*, *Blainvillea*, *Chrysogonum*, *Coreopsis*, *Dahlia*, *Eupatorium*, *Gerbera*, *Helianthus*, *Lagascea*, *Laggera*, *Leucanthemum*, *Mauranthemum* [*Chrysanthemum* s. lat.], *Melampodium*, *Parthenium*, *Telekia*, *Tithonia*, *Xanthium*, *Zinnia*), Fabaceae (*Crotalaria*), Malvaceae (*Abelmoschus*), Polygonaceae (*Persicaria*), Solanaceae (*Solanum*), and Verbenaceae (*Verbena*).

Notes: *Persicaria* species have recently been confirmed as hosts of *G. ambrosiae* by molecular sequence analyses (*P. alpina* [30], Azerbaijan; *P. decipiens* [13], Australia).

***Golovinomyces circumfusus*** (Schltdl.) U. Braun, in Braun & Cook, CBS Biodiversity Series 11: 309 (2012).

≡ *Alphitomorpha circumfusa* Schltdl., Verh. Ges. Naturf. Freunde Berlin 1(1): 49 (1819).

≡ *Erysibe circumfusa* (Schltdl.) Ehrenb., Nova Acta Phys.-Med. Acad. Caes. Leop.-Carol. Nat. Cur. 10: 169 (1821).

≡ *Erysiphe circumfusa* (Schltdl.) Schltdl., Fl. berol. 2: 169 (1824).

≡ *Erysibe circumfusca* (Schltdl.) Link, Sp. pl. 4, 6(1): 109, (1824).

≡ *Erysiphe communis* f. *circumfusa* (Schltdl.) Fr., Syst. mycol. 3: 240 (1829).

= *E. communis* n. *corymbiferarum* Fr., Syst. mycol. 3: 241 (1829), p.p.

= *E. cichoracearum* f. *eupatorii* Dearn., in Rehm, Ascomyc., Fasc. 48, No. 1950 (1911) and Ann. Mycol. 9: 290 (1911).

= *E. cichoracearum* auct. p.p.

= *Golovinomyces cichoracearum* auct. p.p.

*Illustration*: Braun & Cook (2012, p. 309, Fig. 331).

*Literature*: Jaczewski ([57], p. 197).

*Exsiccatae*: Barthol., Fungi Columb. 2930, 4020, 4224, 4919. Rabenh., Klotzschii Herb. Viv. Mycol. 467. Rehm, Ascomyc. 1950. Syd., Mycoth. Germ. 1530.

*Description*: *Mycelium* amphigenous, but sometimes also caulicolous, thin, white, effuse or in distinct patches, persistent on the upper leaf surface and on stems, less conspicuous and often evanescent on lower surface; *hyphae* branched mostly at right angles, hyaline, smooth or almost so, 3–9 μm wide; *hyphal appressoria* usually solitary, slightly to distinctly nipple-shaped, 3–7 μm diam.; *conidiophores* erect, solitary per hyphal mother cell, arising laterally or from the upper surface and usually towards one end of the mother cell, up to 160 μm long, *foot-cells* variable, straight to curved at the base or sinuous, 30–110 × 9–14 μm, almost cylindrical to slightly increasing in width from base to top, occasionally slightly constricted at the 7–9 μm wide basal septum that is usually at the junction with the mother cell or occasionally raised by up to 5 μm, followed by 2–3 shorter cells, forming catenescent conidia; *primary conidia* obovoid, *secondary conidia* ellipsoid-ovoid, subcylindrical, limoniform, 25–40 × 12–20 μm, length/width ratio 1.3–2.6, *germ tubes* terminal or almost so, short to moderately long, slightly clavate, i.e. apex with slightly swollen appressorium, *Euoidium* type. *Chasmothecia* amphigenous and caulicolous, scattered to gregarious, subglobose to somewhat depressed-globose, 85–140 μm diam., rarely larger; *peridium cells* irregularly polygonal, rounded to usually somewhat daedaleoid, 5–25(− 30) μm diam., walls up to 2.5 μm thick; *appendages* numerous, equatorial and in the lower half, mycelioid, simple, rarely branched, (0.25–)0.5–2.5(− 3.5) times as long as the chasmothecial diam., 3–8 μm wide, walls thin (up to 1 μm), smooth to faintly rough, on mature ascomata completely pale to medium dark brown throughout or somewhat paler towards the tip; *asci* numerous, usually 5–15, broad obovoid-saccate or almost globose, (40–)50–70(− 80) × (20–)25–35(− 40) μm, almost sessile to short-stalked, thin-walled, terminal oculus 8–15 μm diam., 2(− 3)-spored; *ascospores* ellipsoid-ovoid, (15–)18–25 × 10–17 μm, colourless.

*Material examined*: all were collected on leaves of *Eupatorium cannabinum*, GERMANY, ex herb. Schlechtendal, without any further data, HAL 1423 F, **lectotype** [designated in Dörfelt & Ali (1987)]; Brandenburg, Landkreis Ostprignitz-Ruppin, Großzerlag, 22 Sep. 2006, H. Boyle, GLM-F74796; Brandenburg, Landkreis Ostprignitz-Ruppin, north-west of Rheinsberg, 24 Sep. 2006, H. Jage, GLM-F85832; Sachsen, Zittau, Westpark, 9 Aug. 2007, H. Boyle, GLM-F80897; Sachsen-Anhalt, Salzwedel, 19 Aug. 2000, H. Jage and H. Lehmann, GLM-F49501; Sachsen-Anhalt, Halle (Saale), Osendorfer See, 12 Nov. 2000, H. Jage (GLM-F47189); Sachsen-Anhalt, Salzwedel, Hoydersburg, 11 Aug. 2004, H. Jage, GLM-F65924. Germany, Brandenburg, Spreewald, Briesensee, 8 Oct. 2016, V. Kummer, HAL 3300 F, **epitype** (designated here, MycoBank MBT385760).

*Host range and distribution*: on *Eupatorium cannabinum* (Asteraceae), Europe (Bulgaria, Czech Republic, Denmark, Finland, France, Germany, Hungary, Italy, Lithuania, Poland, Romania, Russia, Slovakia, Sweden, Switzerland, UK) [58–61].

*Notes:* Braun and Cook [1] assigned *Golovinomyces* on host species belonging to *Eupatorium* s. lat. From the northern hemisphere, including Europe, North America and northern regions of Asia, to *G. circumfusus*. This species seems to be confined to its type host, *E. cannabinum*, as collections from Asian species of *Eupatorium* pertain to *G. ambrosiae*. The affinity and identity of North American collections on *Eupatorium perfoliatum*, *Eutrochium maculatum* (≡ *Eupatorium maculatum*), and *Eutrochium purpureum* (≡ *Eupatorium purpureum*) remain unclear since sequence data and results of detailed morphological examinations of the asexual morphs on these hosts are not yet available. *Golovinomyces* on these hosts is common in North America, including several collections distributed in exsiccatae (Barthol., Fungi Columb. 2930, 4020, 4224, 4919; Rehm, Ascomyc. 1950).

**Fig. 3:**
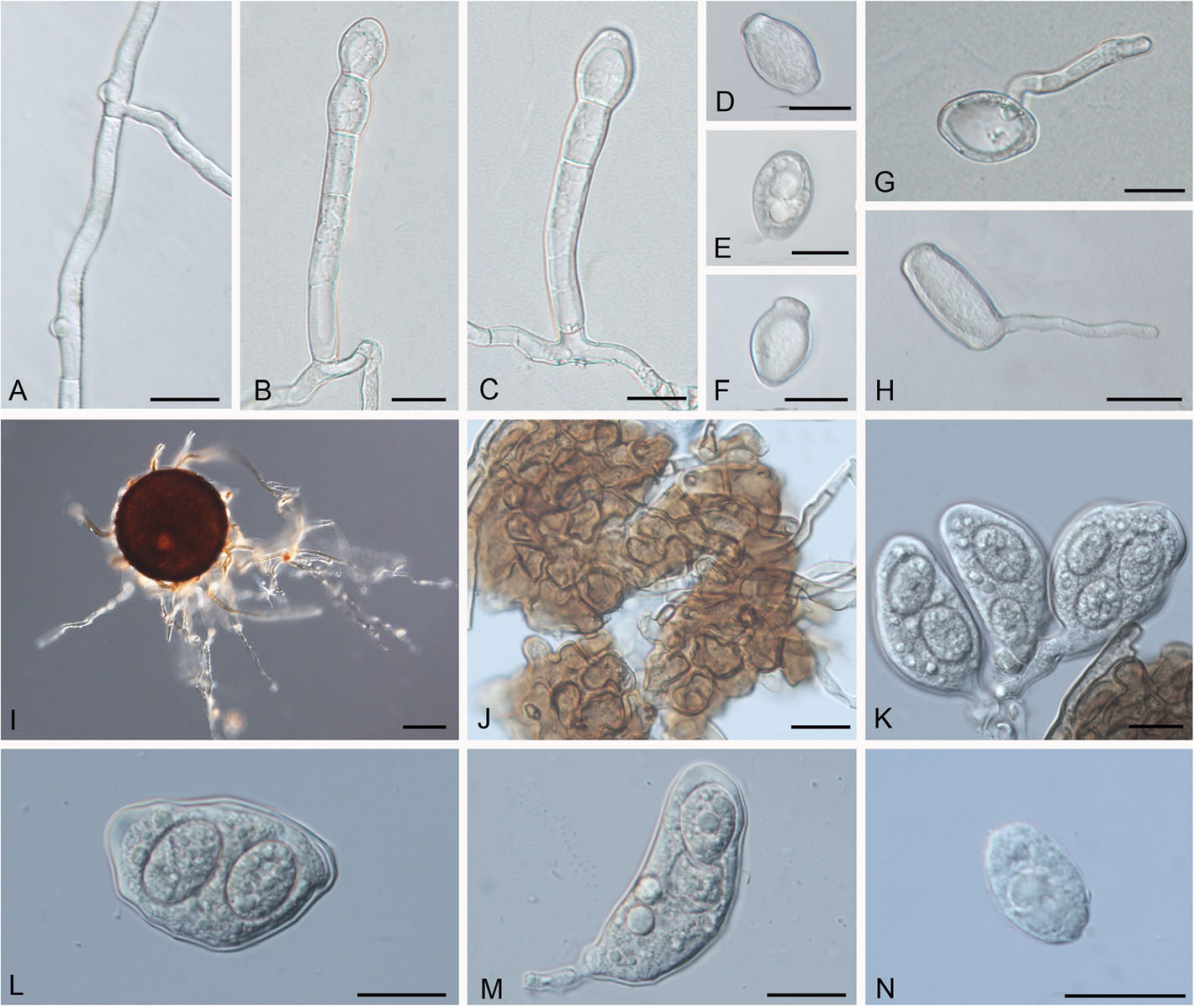
*Golovinomyces latisporus* comb. nov. (HMJAU-PM91828 ex *Helianthus annuus*). **a.** Nipple-shaped hyphal appressorium. **b–c.** Conidiophores. **d–f.** Conidia. **g–h.** Conidial germination. **i.** Chasmothecium. **j.** Peridium cells of Chasmothecium. **k–m.** Asci with two or three ascospores. **n.** Ascospores. Scale bars = 20 μm

***Golovinomyces latisporus*** (U. Braun) P.-L. Qiu & S.-Y. Liu, **comb. nov.** Figure 3.

MycoBank MB 829648.

*Basionym*: *Oidium latisporum* U. Braun, Zentralbl. Mikrobiol. 137: 315 (1982).

= *Erysiphe cichoracearum* f. *helianthi* Jacz., Karm. Opred. Grib., Vip. 2. Muchn.-rosj. Griby (Leningrad): 198 (1927).

= *Erysiphe cichoracearum* var. *latispora* U. Braun, Mycotaxon 18:117 (1983).

≡ *Golovinomyces cichoracearum* var. *latisporus* (U. Braun) U. Braun, Schlechtendalia 3: 51 (1999).

= *E. cichoracearum* auct. p.p.

= *Golovinomyces cichoracearum* auct. p.p.

*Illustrations*: Braun ([62], p. 316, fig. 1 [63];, p. 118, fig. 6 [17];, p. 250, pl. 66, fig. A [58];, p. 270, pl. 40, fig. A), Tanda et al., ([64], p. 254, figs. 1–2), Nomura ([56], p. 185, Fig. 249), Cook and Braun ([65], p. 627, Fig. 5), Chen et al., ([66], p. 4, fig. 1b).

*Description*: *Mycelium* amphigenous, also on stems, effuse or forming patches, thin, white, persistent or almost so; *hyphae* hyaline, walls thin, smooth, 3–8 μm wide; *hyphal appressoria* nipple-shaped, solitary or in opposite pairs, 4–8 μm diam.; *conidiophores* arising centrally or towards one end of hyphal mother cells and from their upper surface, erect, straight, *foot-cells* cylindrical, 35–80 × 9–15 μm, followed by 1–3 shorter cells, forming catenescent conidia; *conidia* broad ellipsoid-ovoid, doliform to somewhat limoniform, 25–45 × 15–27 μm (when fresh), length/width ratio < 2 (1.3–1.9, mostly 1.4–1.6), *germ tubes* dimorphic, with terminal to subterminal, occasionally lateral germination, on glass at 100% RH, long, filiform, up to 10 times as long as the width of the conidium, growing away from the glass surface (negatively hydrotropic), tips not swollen or only slightly swollen when in contact with the surface, = the *longitubus* pattern within the *Euoidium* type, a varying percentage with short, terminal to subterminal germination, subclavate with somewhat swollen apices, especially when they immediately contact a hydrophobic surface, = typical *Euoidium* type. *Chasmothecia* amphigenous, scattered to gregarious, subglobose, (65–)85–130(− 145) μm diam.; *peridium* cells irregularly polygonal to daedaleoid, (5–)10–25(− 35) μm diam., walls up to 2.5 μm wide; *appendages* numerous, arising from the lower half, mycelioid, simple, rarely branched, rarely longer than 0.5–2 times the chasmothecial diam., 3–8 μm wide, septate, walls thin, smooth or almost so, at first colourless, later completely brown or paler towards the tip; *asci* usually 5–15, occasionally more, clavate-saccate, 45–80 × 20–40 μm, short-stalked, wall 1–2 μm wide, 2(− 3)-spored; *ascospores* ellipsoid-ovoid, 18–29 × 9–20 μm, colourless.

*Material examined*: Additional collections used for molecular analyses (see Table 1); GERMANY, Sachsen-Anhalt, Greifenhagen, on leaves of *Helianthus* × *laetiflorus*, 20 Sep. 1981, HAL 1434 F, **holotype** (of *O. latisporum*); Sachsen-Anhalt, Greifenhagen, on leaves of *Helianthus* sp. (cf. *maximiliani*), 20 Sep. 1981, HAL 1443 F, **paratype** (of *O. latisporum*); USA, Wisconsin, Jefferson Co., Faville, Prairie Preserve, near lake Mills, on *Helianthus grosseserratus*, 31 Aug. 1963, H.C. Greene, DAOM 96982, **holotype** (of *E. cichoracearum* var. *latispora*). Switzerland, Vaud, Nyon, on leaves of *Helianthus annuus*, 17 Sep. 2018, HAL 3299 F, **epitype** (designated here, MycoBank MBT385594).

*Host range and distribution*: on *Helianthus* (*angustifolius*, *annuus*, *arizonensis*, *atrorubens*, *californicus*, *carnosus*, *cusickii*, *debilis*, *debilis* subsp. *cucumerifolius*, *decapetalus*, *divaricatus*, *doronicoides*, *exilis*, *giganteus*, *gracilentus*, *grosseserratus*, *hirsutus*, *kellermanii*, ×*laetiflorus*, *laevigatus*, *longifolius*, *maximiliani*, *mexicanus*, *microcephalus*, ×*multiflorus*, *neglectus*, *niveus* subsp. *tephrodes*, *nuttallii*, *nuttallii* subsp. *parishii*, *paradoxus*, *parviflorus*, *pauciflorus*, *pauciflorus* subsp. *strumosus*, *subrhomboideus*, *petiolaris*, *praecox* subsp. *hirtus*, *praecox* subsp. *runyonii*, *radula*, *rigidus*, *salicifolius*, *scaberrimus*, *schweinitzii*, *tuberosus*), *Rudbeckia* (*amplexicaulis*, *bicolor*, *fulgida*, *hirta*, *laciniata*, *occidentalis*, *serotina*, *triloba*), *Zinnia* (*angustifolia*, *elegans*) Asteraceae [Heliantheae]; Africa (South Africa, Tanzania), Asia (China, India, Israel, Japan, Korea, Nepal, Russia [Siberia, Far East], Turkey), Europe (Bulgaria, Germany, Greece, Hungary, Italy, Lithuania, Netherlands, Poland, Romania, Russia, Slovenia, Switzerland, Turkey, Ukraine, former Yugoslavia), North America (Canada, Mexico, USA), South America and West Indies (Argentina, Cuba, Bolivia, Brazil, Chile, Venezuela), Oceania (Fiji, Samoa), New Zealand (see [1, 58, 67], https://nt.ars-grin.gov/fungaldatabases/index.cfm).

*Notes*: *Golovinomyces latisporus* occurs on various *Helianthus* species almost worldwide. *Zinnia angustifolia* and *Z. elegans* are additional hosts proven by means of molecular methods. *Golovinomyces* collections found on various *Rudbeckia* spp. are assigned to *G. latisporus* with respect to the characters of the anamorph, although multilocus sequence analyses are still lacking. Taxonomy of a recently published record of “*G. spadiceus*” on *Helianthus annuus* in the United States [54] is unclear and urgently requires multilocus analyses for species identification. The identity of *Golovinomyces* on *Iva* spp. (*axillaris*, *frutescens*, *xanthifolia*) has not been sufficiently studied.

## Discussion

The taxonomic history of the powdery mildews allied to *Erysiphe cichoracearum* dates back to de Candolle, in Lamarck and de Candolle [68]. He described *E. cichoracearum* on *Scorzonera hispanica* and *Tragopogon porrifolius*. Salmon [14] widened the concept of *E. cichoracearum* considerably by assigning powdery mildew on numerous hosts of various plant families to this species, including *Helianthus* spp. In previous circumscriptions, *E. cichoracearum* was characterized by having ascomata with mycelium-like appendages, several usually 2-spored asci, and conidia formed in chains without fibrosin bodies [14–17]. Braun [62] described the asexual morph of powdery mildew found on *Helianthus* × *laetiflorus* in Germany as *Oidium latisporum* based on the differences in conidial characters (most notably broader conidia) from collections of *E. cichoracearum* on various other hosts. Later, Braun [63] introduced the name *E. cichoracearum* var. *latispora* based on holomorphic North American type material, and cited *E. ambrosiae* as a possible synonym. Heluta [69] reallocated *E. cichoracearum* to *Golovinomyces*. Braun and Cook [1] split *G. cichoracearum* into several species based on molecular analyses of this complex which suggested a co-evolutionary relationship between *Golovinomyces* species and tribes of Asteraceae [18].

*Golovinomyces* on hosts of the Heliantheae was divided into two species, *G. ambrosiae* and *G. spadiceus*, distinguished by clear morphological differences in their asexual morphs [1]. Type material of *E. ambrosiae* was examined, and this name was used for powdery mildew on *Ambrosia*, *Helianthus*, *Iva*, and *Rudbeckia* spp. *E. ambrosiae* was characterized by having broad ellipsoid-ovoid, doliiform to somewhat limoniform conidia, 25–45 × 15–27 μm (when fresh) with a length/width ratio < 2 (1.3–1.9, mostly 1.4–1.6), and dimorphic germ tubes that were long and filiform (longitubus pattern with the *Euoidium* conidial germination type) and consisted of a varying percentage of shorter germ tubes that were often swollen at the tip (ordinary *Euoidium* germ tubes) [1]. Whereas, the conidial shape and size, as well as the conidial germination pattern of *G. spadiceus* agrees with the common *Euoidium* type. These morphological differences were not reflected in a comprehensive phylogenetic analyses based on ITS and 28S rDNA powdery mildews previously referred to as *G. ambrosiae* and *G. spadiceus.* In the phylogenetic analyses, *G. ambrosiae* and *G. spadiceus* formed a single undifferentiated clade (lineage III in Takamatsu et al., [19]). Furthermore, this clade also encompassed sequences obtained from *Golovinomyces* on *Eupatorium chinense* in Japan [referred to as *G. circumfusus* based on the circumscription of this species in Braun and Cook [1] and the assumption that all *Golovinomyces* collections on various *Eupatorium* species in Asia, Europe and North America pertain to a single species] as well as sequences from *Golovinomyces* on numerous Asteraceae hosts from several tribes and even other families. The extensive host range exhibited by clade 3 suggests the involvement of a plurivorous species.

Sequences from the five gene regions could not be obtained for all samples used in this study. The phylogenetic affinity of *G. circumfusus* could be clarified by the inclusion of sequences obtained from powdery mildew on *E. cannabinum* (type host) in Germany (type region). *G. circumfusus* on its type host does not cluster within the former “Heliantheae Clade” and is not closely allied with *G. ambrosiae* complex. It represents a well-supported species of its own, confined to *E. cannabinum* in Europe. Blumer ([16], p. 188) summarized results of previous inoculation tests carried out by himself and other authors and classified *Erysiphe cichoracearum* s. lat. on *E. cannabinum* as a biologically specialized form (f. sp. *eupatorii*), confined to this host. In order to stabilize the application of the old name *Erysiphe circumfusa*, described in the nineteenth century, an epitype has been designated. Powdery mildew on Asian *Eupatorium* spp. is not conspecific with *G. circumfusus* and pertains to a clade previously referred to as *G. spadiceus* [13]. This clade represents a plurivorous species on a wide range of hosts belonging to the Heliantheae and other tribes of Asteraceae as well as hosts of other plant families. However, the naming of this clade had to be corrected.

Sequences from *Golovinomyces* on *Ambrosia* spp. in Asia and North America do not cluster together with sequences obtained from *Golovinomyces* on *Helianthus* spp., but they pertain to the former plurivorous *G. spadiceus*. The morphological characters of the powdery mildew on *Ambrosia* also agree with that of *G. spadiceus* (the type material of *Erysiphe ambrosiae* contains chasmothecia, but the features of the asexual morph could not be properly examined). Hence, Braun [63] cited *E. ambrosiae* as a potential synonym of *E. cichoracearum* var. *latispora*. The application of the name *E. ambrosiae* in Braun and Cook [1], based on this questionable synonymy, must be classified as a misinterpretation. These results have nomenclatural and taxonomic consequences, viz., the older name *Erysiphe ambrosiae*, which has priority over *G. spadiceus*, is now the correct name for this plurivorous species, and *G. spadiceus* and its synonyms must be reduced to synonymy with *G. ambrosiae*. Finally, *Golovinomyces* on *Helianthus* spp., morphologically distinguished from the former *G. spadiceus*, turned out be genetically different as well (although undoubtedly closely allied to the latter species).

Since *G. ambrosiae* now represents an older name for the species previously referred to as *G. spadiceus*, it is necessary to rename the species on *Helianthus*. Hence, *Oidium latisporum* (= *Erysiphe cichoracearum* var. *latispora*), the oldest valid name for this taxon at the species level, is used as the basionym for the combination *G. latisporus*. This species is common with a near global distribution, and also occurs on *Zinnia* [sequences retrieved from *Z. angustifolia* (HAL 2338 F) refer to a German collection from a botanical garden in which the *Zinnia* grew close to *Helianthus* plants infected by *G. latisporus*]. Sequences retrieved from *Z. elegans* (HMJAU-PM91850) refer to a collection from the Sichuan province of China where no *Helianthus* plants grew. The powdery mildew on *Rudbeckia* coincides morphologically with *G. latisporus*. However, currently only ITS and 28S sequences are available [19]. Future examinations based on IGS, *TUB2* and *CHS1* are necessary to confirm the identity. In any case, the example of *Zinnia* shows that host plants of other genera, such as *Helianthus* or *Iva*, might also be infested by the two closely allied species, *G. ambrosiae* and *G. latisporus*. In order to answer this question, a combination of morphological examinations and phylogenetic analyses based on a multilocus approach are required in the future.

## Conclusions

The phylogenetic analyses of multilocus sequence data, including ITS and 28S rDNA, IGS, *TUB2*, *CHS1*, and consideration of morphological characters enabled to resolve species delimitation in a heterogeneous complex within the genus *Golovinomyces*. The old names involved in this complex have been epitypified, providing ex-epitype sequence data, and three species were distinguished in the complex named *G. ambrosiae* emend. (including *G. spadiceus*), *G. latisporus* comb. nov. (≡ *Oidium latisporum*), and *G. circumfusus* confined to *Eupatorium cannabinum* in Europe. This research illustrated that such approaches are suitable and promising in cases of phylogenetically closely allied assemblages of powdery mildew species in which ITS analyses do not yield sufficient resolution.

## Supplementary information

**Additional file 1: Figure S1.** Phylogenetic analysis of the ITS region of the *Golovinomyces ambrosiae* complex and *G. circumfusus*. The tree was constructed based on 73 sequences from tribe Golovinomyceteae. One sequence from *Erysiphe kenjiana* (accession number: MK452611) was used as an outgroup. Bootstrap values based on 1000 replications are indicated above the branches.

**Additional file 2: Figure S2.** Phylogenetic analysis of the 28S rDNA region of the *Golovinomyces ambrosiae* complex and *G. circumfusus*. The tree was constructed based on 74 sequences from tribe Golovinomyceteae. One sequence from *Erysiphe kenjiana* (accession number: MK452684) was used as an outgroup. Bootstrap values based on 1000 replications are indicated above the branches.

**Additional file 3: Figure S3.** Phylogenetic analysis of the IGS region of the *Golovinomyces ambrosiae* complex and *G. circumfusus*. The tree was constructed based on 74 sequences from tribe Golovinomyceteae. Three sequences from *Neoërysiphe galeopsidis* (accession numbers: MK452530, MK452531, MK452532) were used as an outgroup. Bootstrap values based on 1000 replications are indicated above the branches.

**Additional file 4: Figure S4.** Phylogenetic analysis of *Golovinomyces ambrosiae* complex and *G. circumfusus* based on the *TUB2* region. The tree was constructed based on 43 sequences from tribe Golovinomyceteae and one sequence from *Erysiphe kenjiana* (accession number: MK452458) was used as an outgroup. Bootstrap values based on 1000 replications are indicated above/below the branches.

**Additional file 5: Figure S5.** Phylogenetic analysis of the *CHS1* region of the *Golovinomyces ambrosiae* complex and *G. circumfusus*. The tree was constructed based on 49 sequences from tribe Golovinomyceteae. Three sequences from *Neoërysiphe galeopisidis* (accession numbers: MK452438, MK452439, MK452440) was used as an outgroup. Bootstrap values based on 1000 replications are indicated above/below the branches.

## Data Availability

The molecular data in the manuscript can be found in the GenBank database after publishing, and the materials can be found in the Herbaria shown in Table 1.

## Abbreviations

auct: Auctorum (in the sense of other authors)
comb. nov: Combinatio nova (new combination)
diam: Diameter
e.g.: For example
emend: Emendation
f. sp.: Forma specialis
Figs: Figures
No: Number
P: Page
p.p: Pro parte (partly)
s: Sensu
s. lat: Sensu lato (in the broad sense)
spp.: Species plural
var.: Variety
viz.: Namely
