# Supplementary figures and images for: Multi-locus phylogeny and taxonomy of an unresolved, heterogeneous species complex within the genus Golovinomyces (Ascomycota, Erysiphales), including G. ambrosiae, G. circumfusus and G. spadiceus
Source: BMC Microbiol. 2020 Mar 5;20:51. doi: 10.1186/s12866-020-01731-9 (PMC7059721; doi:10.1186/s12866-020-01731-9)

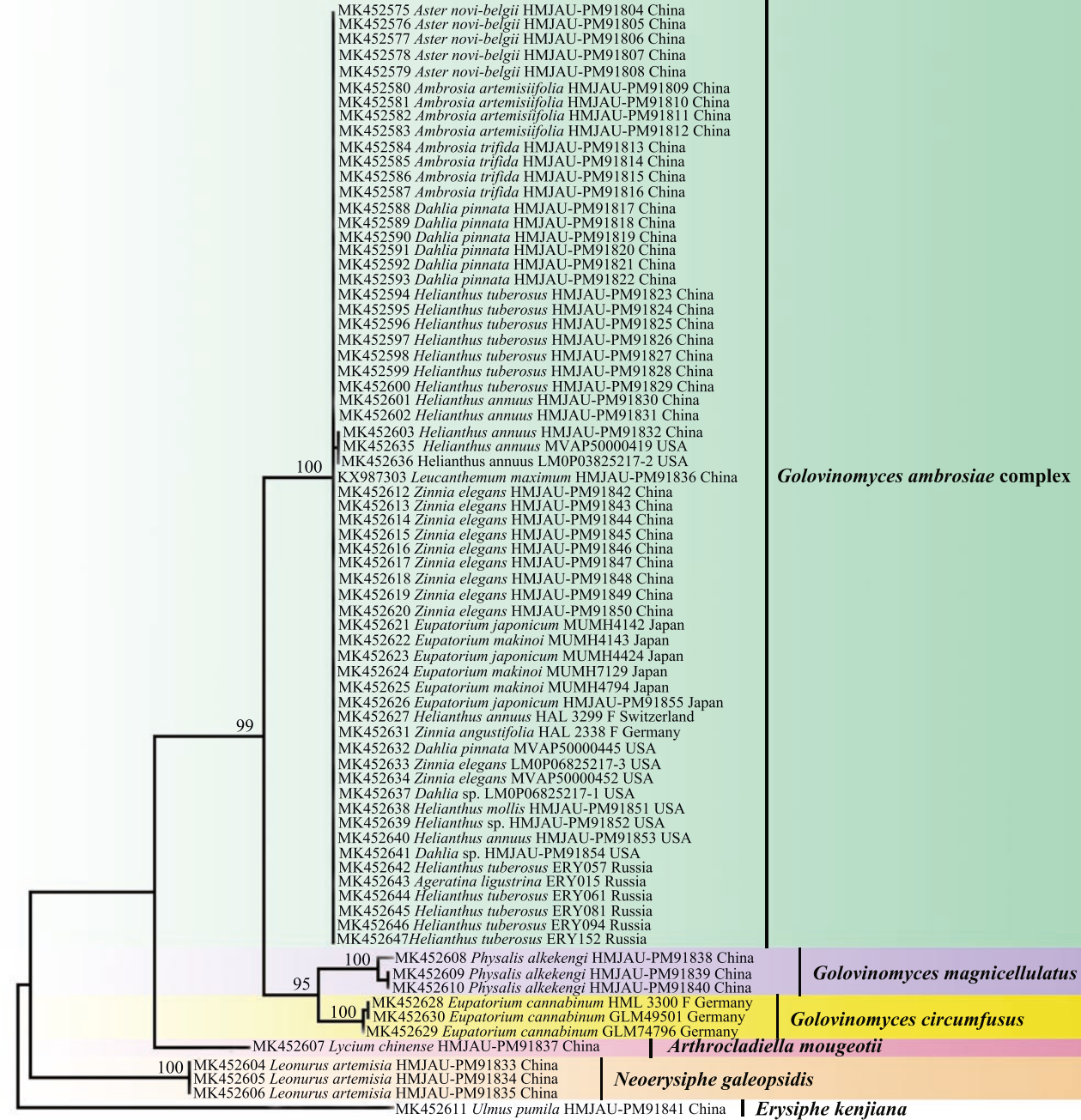

Supplement: Supplementary file 1 — Additional file 1: Figure S1. Phylogenetic analysis of the ITS region of the Golovinomyces ambrosiae complex and G. circumfusus. The tree was constructed based on 73 sequences from tribe Golovinomyceteae. One sequence from Erysiphe kenjiana (accession number: MK452611) was used as an outgroup. Bootstrap values based on 1000 replications are indicated above the branches. [file 12866_2020_1731_MOESM1_ESM.pdf]

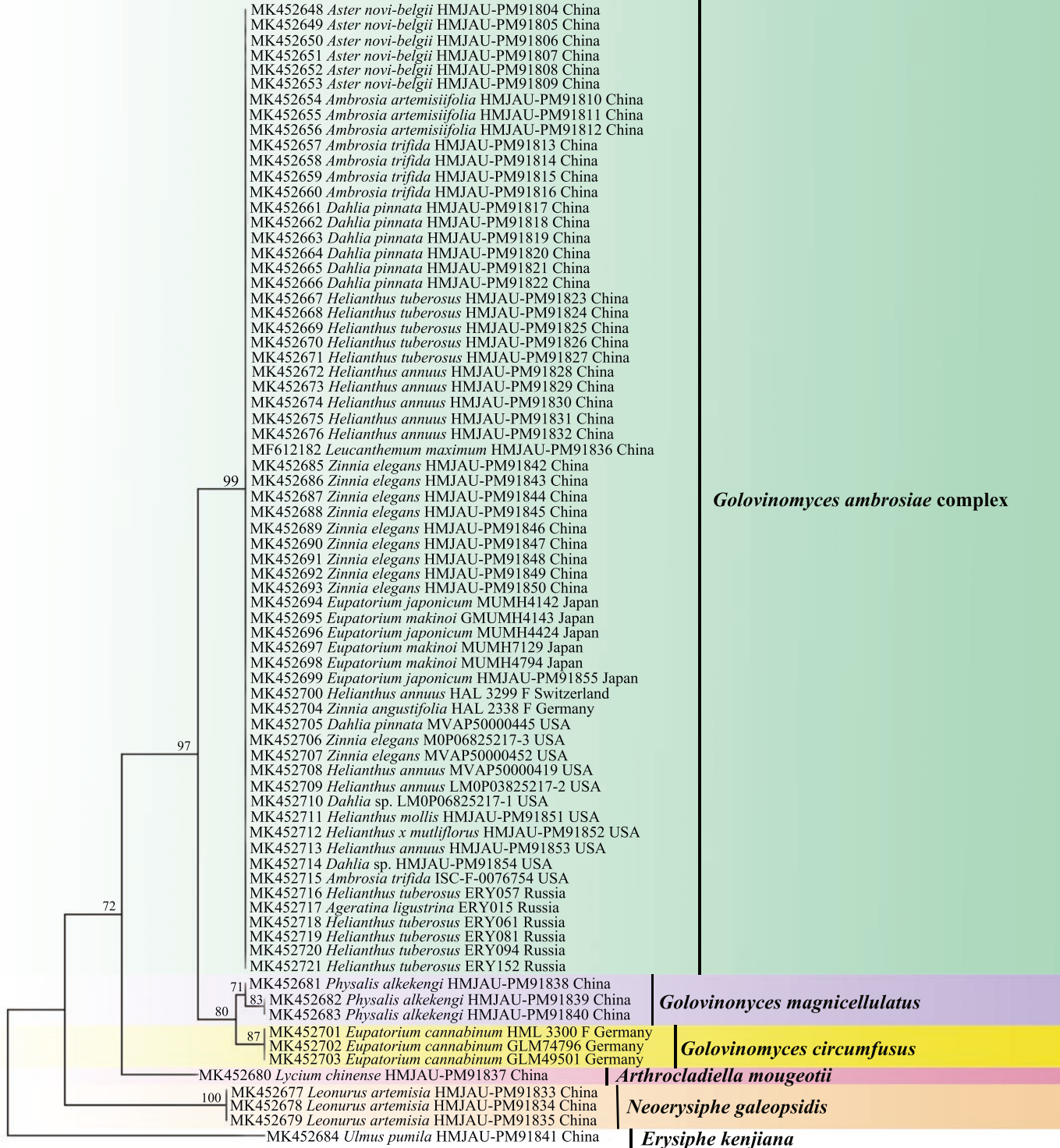

Supplement: Supplementary file 2 — Additional file 2: Figure S2. Phylogenetic analysis of the 28S rDNA region of the Golovinomyces ambrosiae complex and G. circumfusus. The tree was constructed based on 74 sequences from tribe Golovinomyceteae. One sequence from Erysiphe kenjiana (accession number: MK452684) was used as an outgroup. Bootstrap values based on 1000 replications are indicated above the branches. [file 12866_2020_1731_MOESM2_ESM.pdf]

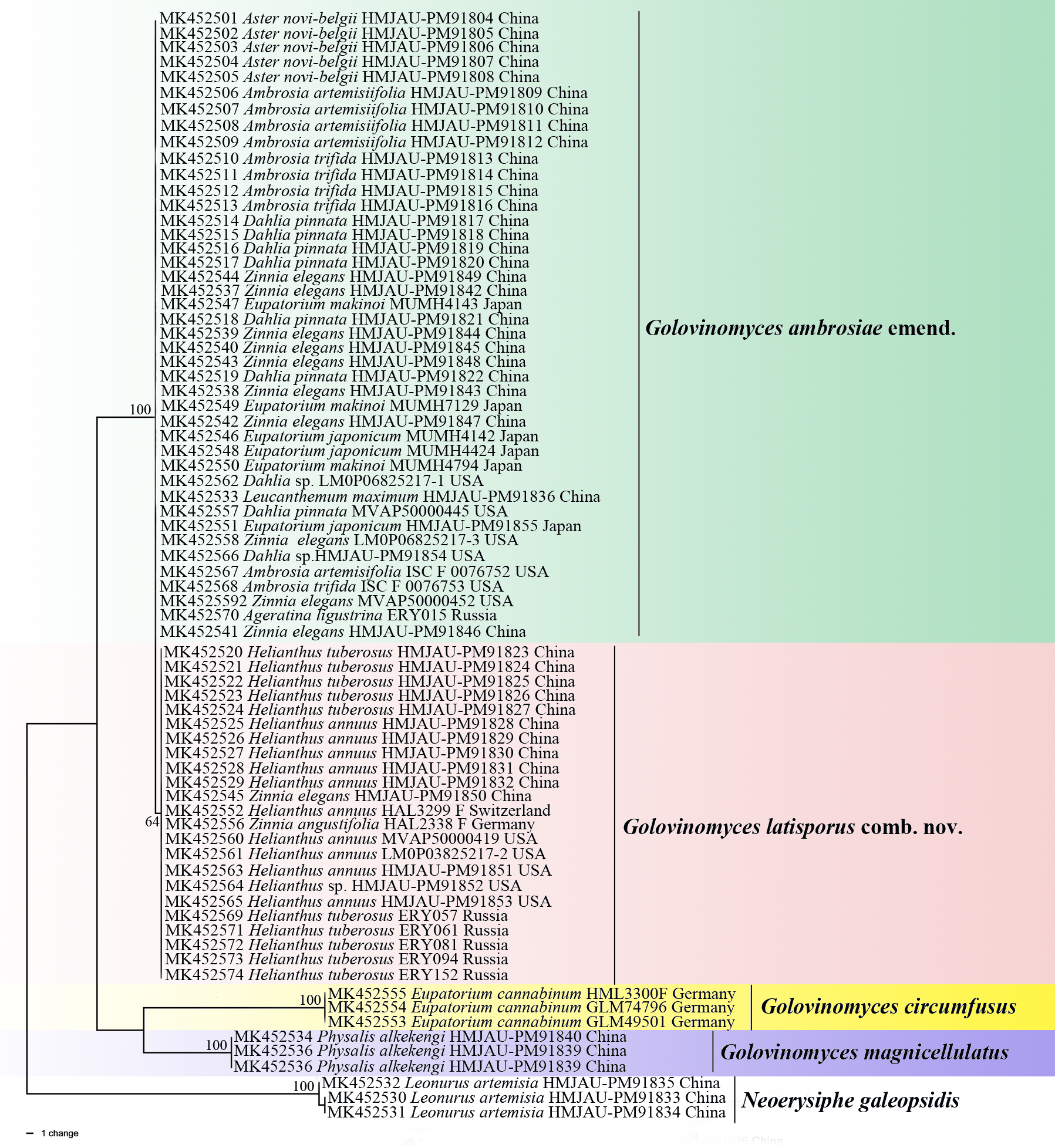

Supplement: Supplementary file 3 — Additional file 3: Figure S3. Phylogenetic analysis of the IGS region of the Golovinomyces ambrosiae complex and G. circumfusus. The tree was constructed based on 74 sequences from tribe Golovinomyceteae. Three sequences from Neoërysiphe galeopsidis (accession numbers: MK452530, MK452531, MK452532) were used as an outgroup. Bootstrap values based on 1000 replications are indicated above the branches. [file 12866_2020_1731_MOESM3_ESM.tif]

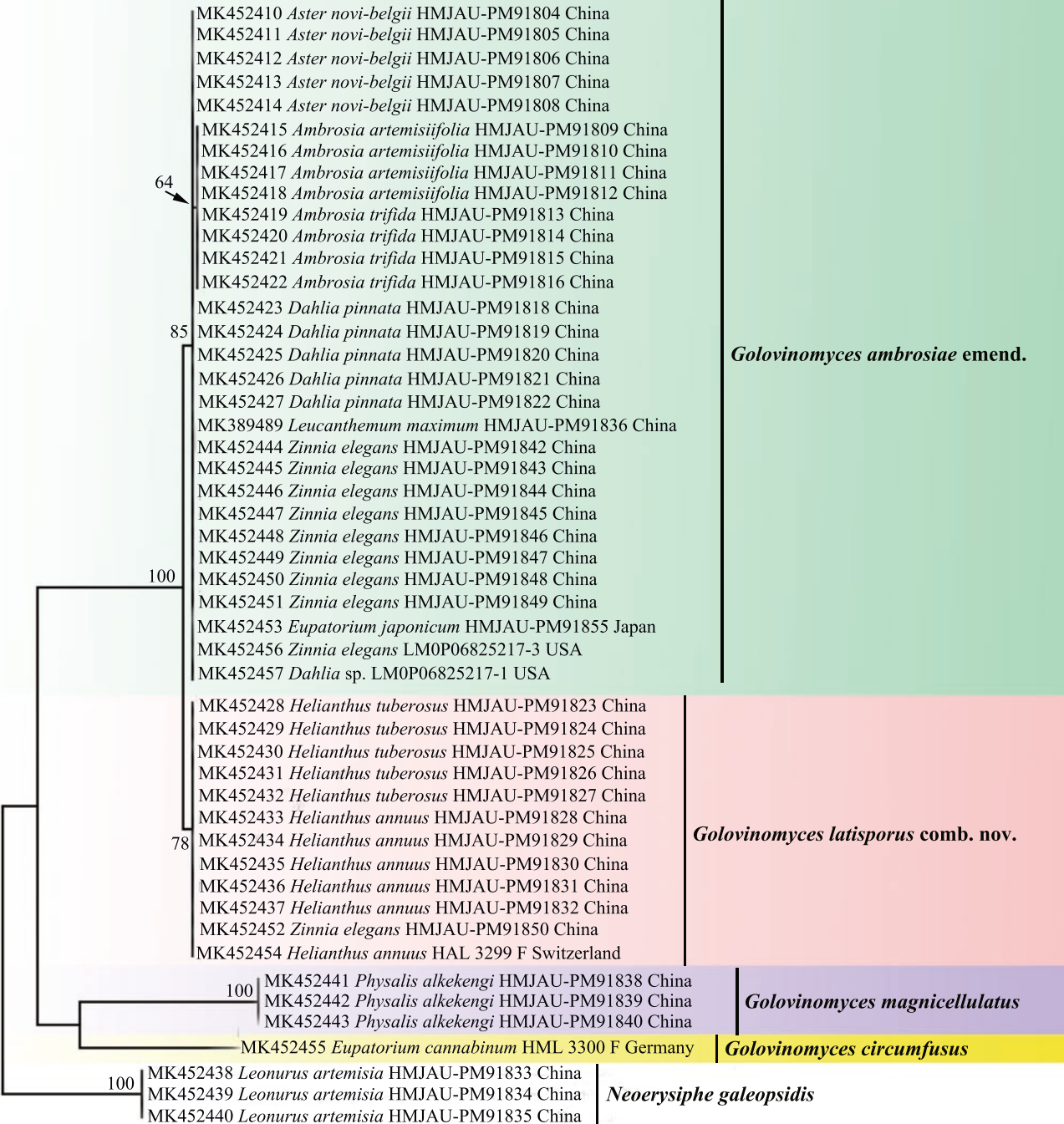

Supplement: Supplementary file 5 — Additional file 5: Figure S5. Phylogenetic analysis of the CHS1 region of the Golovinomyces ambrosiae complex and G. circumfusus. The tree was constructed based on 49 sequences from tribe Golovinomyceteae. Three sequences from Neoërysiphe galeopisidis (accession numbers: MK452438, MK452439, MK452440) was used as an outgroup. Bootstrap values based on 1000 replications are indicated above/below the branches. [file 12866_2020_1731_MOESM5_ESM.pdf]
