# Supplementary material for: Multi-locus phylogeny and taxonomy of an unresolved, heterogeneous species complex within the genus Golovinomyces (Ascomycota, Erysiphales), including G. ambrosiae, G. circumfusus and G. spadiceus
Source: BMC Microbiol. 2020 Mar 5;20:51. doi: 10.1186/s12866-020-01731-9 (PMC7059721; doi:10.1186/s12866-020-01731-9)
Supplement: Supplementary file 4 — Additional file 4: Figure S4. Phylogenetic analysis of Golovinomyces ambrosiae complex and G. circumfusus based on the TUB2 region. The tree was constructed based on 43 sequences from tribe Golovinomyceteae and one sequence from Erysiphe kenjiana (accession number: MK452458) was used as an outgroup. Bootstrap values based on 1000 replications are indicated above/below the branches. [file 12866_2020_1731_MOESM4_ESM.pdf]

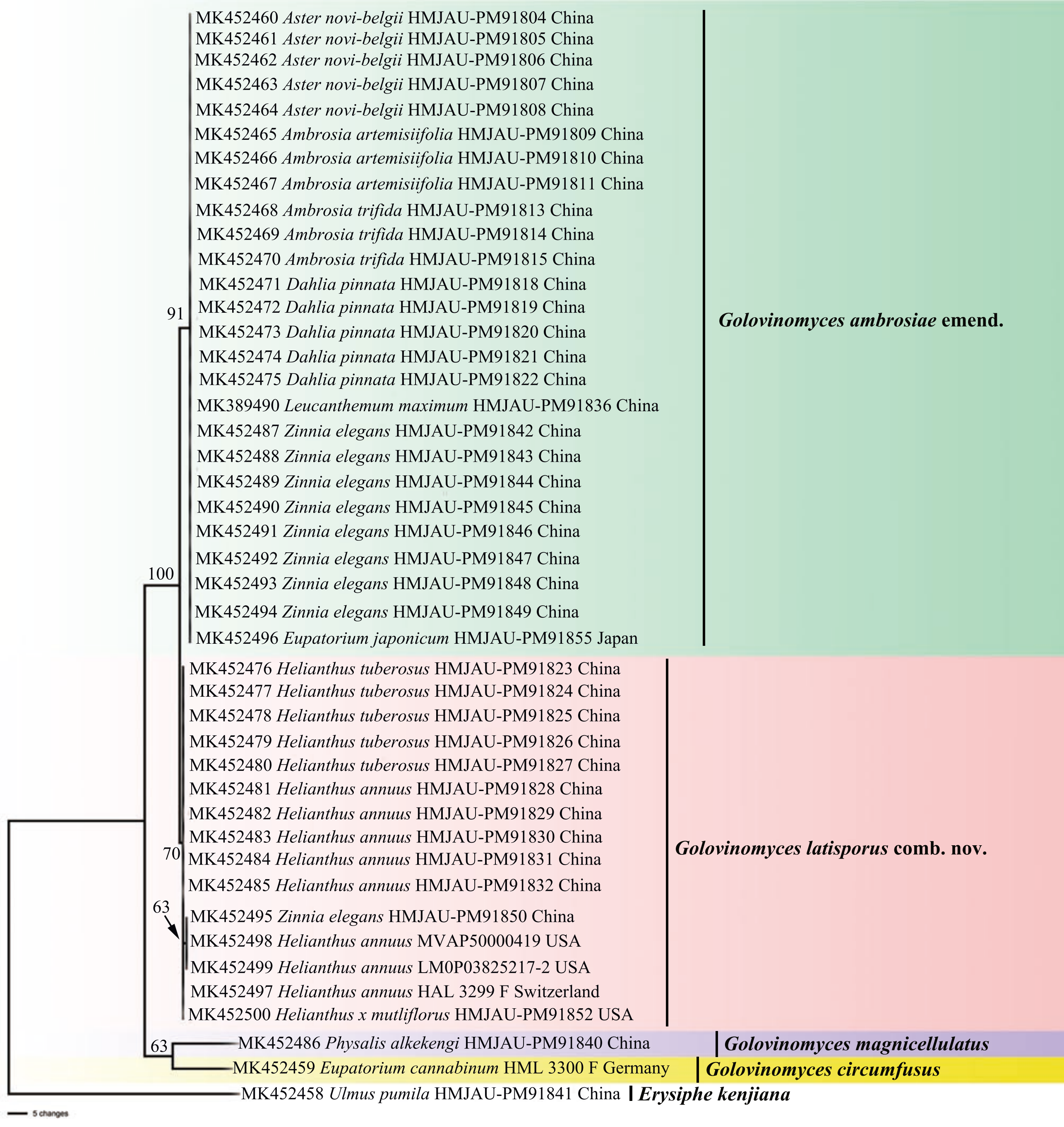
MK452460 Aster novi-belgii HMJAU-PM91804 China
MK452461 Aster novi-belgii HMJAU-PM91805 China
MK452462 Aster novi-belgii HMJAU-PM91806 China
MK452463 Aster novi-belgii HMJAU-PM91807 China
MK452464 Aster novi-belgii HMJAU-PM91808 China
MK452465 Ambrosia artemisiifolia HMJAU-PM91809 China
MK452466 Ambrosia artemisiifolia HMJAU-PM91810 China
MK452467 Ambrosia artemisiifolia HMJAU-PM91811 China
MK452468 Ambrosia trifida HMJAU-PM91813 China
MK452469 Ambrosia trifida HMJAU-PM91814 China
MK452470 Ambrosia trifida HMJAU-PM91815 China
MK452471 Dahlia pinnata HMJAU-PM91818 China
MK452472 Dahlia pinnata HMJAU-PM91819 China
MK452473 Dahlia pinnata HMJAU-PM91820 China
MK452474 Dahlia pinnata HMJAU-PM91821 China
MK452475 Dahlia pinnata HMJAU-PM91822 China
MK389490 Leucanthemum maximum HMJAU-PM91836 China
MK452487 Zinnia elegans HMJAU-PM91842 China
MK452488 Zinnia elegans HMJAU-PM91843 China
MK452489 Zinnia elegans HMJAU-PM91844 China
MK452490 Zinnia elegans HMJAU-PM91845 China
MK452491 Zinnia elegans HMJAU-PM91846 China
MK452492 Zinnia elegans HMJAU-PM91847 China
MK452493 Zinnia elegans HMJAU-PM91848 China
MK452494 Zinnia elegans HMJAU-PM91849 China
MK452496 Eupatorium japonicum HMJAU-PM91855 Japan
Golovinomyces ambrosiae emend.
91
100
MK452476 Helianthus tuberosus HMJAU-PM91823 China
MK452477 Helianthus tuberosus HMJAU-PM91824 China
MK452478 Helianthus tuberosus HMJAU-PM91825 China
MK452479 Helianthus tuberosus HMJAU-PM91826 China
MK452480 Helianthus tuberosus HMJAU-PM91827 China
MK452481 Helianthus annuus HMJAU-PM91828 China
MK452482 Helianthus annuus HMJAU-PM91829 China
MK452483 Helianthus annuus HMJAU-PM91830 China
MK452484 Helianthus annuus HMJAU-PM91831 China
MK452485 Helianthus annuus HMJAU-PM91832 China
MK452495 Zinnia elegans HMJAU-PM91850 China
MK452498 Helianthus annuus MVAP50000419 USA
MK452499 Helianthus annuus LM0P03825217-2 USA
MK452497 Helianthus annuus HAL 3299 F Switzerland
MK452500 Helianthus x mutliflorus HMJAU-PM91852 USA
Golovinomyces latisporus comb. nov.
70
63
MK452486 Physalis alkekengi HMJAU-PM91840 China
Golovinomyces magnicellulatus
63
MK452459 Eupatorium cannabinum HML 3300 F Germany
Golovinomyces circumfusus
MK452458 Ulmus pumila HMJAU-PM91841 China
Erysiphe kenjiana
5 changes
